# Programmed death-ligand 1 (PD-L1) is expressed in a significant number of the uterine cervical carcinomas

**DOI:** 10.1186/s13000-017-0631-6

**Published:** 2017-06-17

**Authors:** Opal L. Reddy, Peter I. Shintaku, Neda A. Moatamed

**Affiliations:** Department of Pathology and Laboratory Medicine, David Geffen School of Medicine at UCLA, 10833 Le Conte Avenue, BOX 951732, 1P-241 CHS, Los Angeles, CA 90095-1732 USA

**Keywords:** Uterine cervix, Cervical cancer, Squamous cell carcinoma, Endocervical adenocarcinoma, Adenosquamous carcinoma, PD-L1, Immunohistochemistry, Immunotherapy, Tissue microarray

## Abstract

**Background:**

The programmed death-1/programmed death-ligand-1 (PD-1/PD-L1) immune regulatory axis has emerged as a promising new target for cancer therapeutics, with lasting responses seen in the treatment of metastatic renal and lung carcinomas, as well as melanomas. As tumor surface expression of PD-L1 has been found to correlate with objective responses to anti-PD-L1 immunotherapies, we investigated the expression of PD-L1 in human cervical tumors and provide an adopted scoring system for the systematic evaluation of PD-L1 staining.

**Methods:**

Immunohistochemical staining for PD-L1 expression was performed on a tissue microarray of 101 normal and neoplastic cervical tissues. Neoplastic cores were divided into three groups: squamous cell carcinoma, adenosquamous carcinoma, and endocervical adenocarcinoma. PD-L1 expression was scored based on an adopted scoring system accounting to percentage and intensity of positivity, and results provided alongside available clinical and demographic data.

**Results:**

Overall, PD-L1 was positive in 32 of 93 (34.4%) cervical carcinomas. Subcategorically, PD-L1 was positive in 28 of 74 (37.8%) squamous cell carcinomas, two of seven (28.6%) adenosquamous carcinomas, and two of 12 (16.7%) endocervical adenocarcinomas. It was negative in six benign cervical tissues.

**Conclusions:**

This study shows a significant expression of PD-L1 in 34.4% of cervical carcinomas and no expression of PD-L1 in benign cervical tissues. These findings suggest a role for further investigation of anti-PD-L1/PD-1 immunotherapies in the treatment of PD-L1-positive cervical tumors. In addition, our adopted scoring system will facilitate more systematic correlations between tumor reactivity and response to treatment.

This work was presented at the annual meeting of the United States and Canadian Academy of Pathology (USCAP) in Seattle Washington on March 14, 2016 [1].

## Background

Cancer of the uterine cervix is the third most common gynecologic cancer in the United States, with approximately 13,000 new cases and 4120 cancer deaths estimated to occur in 2016 [2]. Persistent human papillomavirus (HPV) infection, particularly with HPV16, HPV18, and other high risk types, plays a key role in the development of cervical cancer. While screening measures for cervical dysplasia have decreased the incidence of cervical cancer in the United States, cervical cancer remains a major world health problem for women [3, 4]. Depending on the disease stage, treatment for cervical cancer may involve a combination of hysterectomy, pelvic lymph node dissection, and chemoradiation. Targeted therapies using small molecules or monoclonal antibodies are largely still in clinical trial stages [4].

PD-1 is an immune suppressive molecule in the B7-CD28 family that regulates T-cell activation [5]. PD-L1 is a transmembrane protein that can be expressed on tumor cells in the cancerous microenvironment [6]. PD-L1 has been hypothesized to bind its receptor PD-1 on T-cells to downregulate anti-tumor T-cell activity and facilitate immune evasion [7]. Expression of PD-L1 has also been found to be associated with worse survival in solid tumors, including esophageal, gastric, colorectal cancers, and pulmonary adenocarcinoma [8, 9].

The PD-1/PD-L1 axis has emerged as a promising new target for immune checkpoint therapies. Lasting responses have been seen with anti-PD-L1 antibodies in the treatment of metastatic renal and lung carcinomas, as well as melanomas [7]. Currently, there are also ongoing clinical phase I/II trials evaluating the effects of anti-PD-1 therapies, including pembrolizumab (NCT02054806) and nivolumab (NCT02488759), against advanced cervical cancer, though no study results have been reported [10]. Tumor surface expression of PD-L1 has been found to correlate with objective responses to anti-PD-L1 immunotherapies, and provide a rationale for screening tumor samples to identify candidates for these targeted therapies [11]. As data is still lacking on the expression patterns of PD-L1 in benign and malignant cervical tissues, we investigated the expression of PD-L1 in human cervical tissue and cervical tumors on tissue microarrays. Our findings provide rationale for further investigation of PD-L1 immunotherapies in the treatment of cervical cancer. Since, currently there is no unified scoring system for PD-L1 expression by immunohistochemistry as opposed to Her2/neu [12], we are introducing a scoring algorithm which is based on the work published by Garon et al. [13].

## Methods

This study was approved by the Office of the Research Protection Program at University of California, Los Angeles (IRB# 15-001035-CR-00001).

### Tissue microarray

We obtained glass slides of human formalin-fixed paraffin embedded cervical tissue microarray (TMA) from Abcam Inc. (Cambridge, MA) [14]. The TMA included approximately 100 cores of both normal and neoplastic cervical tissues. Hematoxylin and eosin staining was performed on one slide for histopathology evaluation. Diagnoses of the cores were reevaluated by one of the authors (NAM), a gynecopathologist, for diagnostic and grading accuracy. Histopathologic diagnoses were made per established criteria and nomenclature [15–17]. Per the Abcam specifications, all tissues were fixed in 10% neutral buffered formalin for 24 h and processed using identical standard operating procedures. Sections were freshly cut upon order and placed on Superfrost-Plus or Starfrost adhesive slides [14]. Upon examination, the sections were consistent with 4–6 μm in thickness.

### Immunohistochemical staining and intensity

**Fig. 1:**
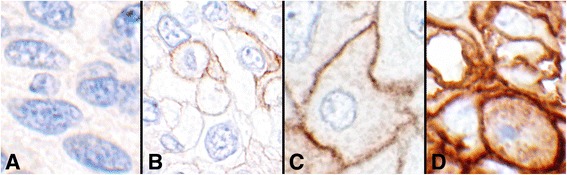
Intensities of the PD-L1 reactions (objective 60×). **a** Intensity 0, showing no cell membrane reaction (#8, Table 2). **b** Intensity 1+, showing weak and/or incomplete circumferential cell membrane reactions (#16, Table 2). **c** Intensity 2+, showing distinct complete circumferential cell membrane staining (#7, Table 1). **d** Intensity 3+, showing very strong circumferential cell membrane reaction (#47, Table 2)

Rabbit anti-human PD-L1 monoclonal antibody (clone SP142) was obtained from Spring Bioscience (Pleasanton, CA) [18]. Ventana Medical Systems, Inc. (Tucson, AZ) has based its assay on this clone [19]. Immunohistochemistry (IHC) was carried out on one of the TMA slides employing the anti-PD-L1 antibody at a 1:100 dilution, adhering to the general guidelines recommended by the Spring Bioscience protocol [18]. Appropriate positive and negative controls were included. The results were recorded based on the intensity of the staining reaction on the cell membranes as described below (Fig. 1), as well as the estimated percentage of positive tumor cells. Positive non-epithelial cells as well as cytoplasmic reactions were discounted.

Intensity 0: If there was no reaction on the cell membranes (Fig. 1a).

Intensity 1+: If the reaction was barely visible or was partially circumferential (Fig. 1b).

Intensity 2+: If the reaction was clearly visible and was completely circumferential (Fig. 1c).

Intensity 3+: If the reaction was intense and fully circumferential (Fig. 1d).

### Statistical analysis

Statistical analysis was performed using a 2 × 2 table for nonparametric Fisher Exact testing. A two-sided *p*-value of 0.05 or less was considered a significant difference between the two compared sets of data. Office-365 Excel (Microsoft, Redmond, WA) and Statistica (version 12; StatSoft Inc, Tulsa, OK) were used for tabulation of data and the statistical analyses [20].

### Study design

A PD-L1 scoring scheme was adopted from Garon et al. for anti-PD-1 treatment in non-small cell lung carcinomas (NSCLC) [13]. Based on their protocol developed during the initial trial phase, all lung tumor biopsies with 50% or more cells showing the PD-L1 reaction, regardless of the intensity, were considered as “positive” for the immunotherapy purposes as it is a practice in our institution [13]. In our adopted scoring system, we evaluated the non-necrotic cells in normal and neoplastic tissues resulting in three categories, “Negative”, “Low-Positive (LoPos)”, and “Positive” as defined in the scoring scheme below:

- *Score “* ***0*** *”*–Positive cells: **0%**; Intensity: **0**; Interpretation: ***Negative***.
- *Score “* ***1a*** *”*–Positive cells: **<50%**; Intensity: **1+**, Interpretation: ***Low-Positive***.
- *Score “* ***1b*** *”*–Positive cells: **<50%**; Intensity: **2+** and/or **3+**; Interpretation: ***Low-Positive***.
- *Score “* ***2a*** *”*–Positive cells: **≥50%**; Intensity: **1+**; Interpretation: ***Positive***.
- *Score “* ***2b*** *”*–Positive cells: **≥50%**; Intensity: **2+** and/or **3+**; Interpretation: ***Positive***.

If all cells had no PD-L1 reaction, a score of “0” and the interpretation of “Negative” were assigned. If 50% or more of the cells had the PD-L1 reactions, a score of “2” and the interpretation of “Positive” was given. The “Positive” interpretation had two subsets, “2a” if the intensity was 1+ and “2b” if the intensity was either 2+ or 3+ (see above). If there was ≥1% but <50% cellular reactivity a score of “1” and an interpretation of “Low-Positive” were assigned with two subsets of “1a” and “1b”. Similar to the “Positive” interpretation, “1a” and “1b” scorings were for 1+, and 2+ or 3+ intensities respectively.

Based on the histopathological diagnoses, the core samples were divided into three groups: **Group I**, squamous cell carcinoma; **Group II**, adenosquamous carcinoma; and **Group III**, endocervical adenocarcinoma. Using the designed scoring method, PD-L1 expression findings were recorded for each group & subgroup and tabulated in their respective tables. The available corresponding clinical and demographic information was extracted from the Abcam product booklet and added alongside the findings [14].

## Results

Of the 101 cores of uterine cervical tissues on the TMA slide, two were excluded for technical issues of being partially or completely washed off during the staining procedures. Six of the cores were benign, with three normal cervical samples and three cervical tissues with cervicitis. All the six benign cervical samples were histopathologically verified and interpreted as “Negative” for PD-L1 expression. The remaining 93 cores were carcinomas, sampled from a patient population with a median age of 44 years. Thirty-two (34.4%) of these samples were “Positive” and 25 (26.9%) were “Negative” for the PD-L1 reactivity. In addition, twelve (12.9%) cores had 1+ (“LoPos, 1a”) reactivity and Twenty-four (25.8%) cores had 2+ or 3+ (“LoPos, 1b”) reactivity for PD-L1 in less than 50% of the tumor cells, a total of 38.7%.

### Group I, squamous cell carcinoma

Of the 93 cores, 74 were squamous cell carcinomas (SCCs). The patients’ median age was 44.5 years old. Among these tumors, 28 were “Positive” for the PD-L1 reactions. This group was further divided into three subgroups based on the histopathological grading of the tumors.

### Subgroup IA, SCC Grade-I

This subgroup comprised of seven core samples with patients’ ages ranging from 37 to 68, with a median of 46 years old. Three (42.9%) of the cases had “Positive” PD-L1 expression, all with the score of “2b”. There were one “Negative”, one “LoPos, 1a”, and two “LoPos, 1b” cores constituting the remaining four (Table 1).

**Table 1:**
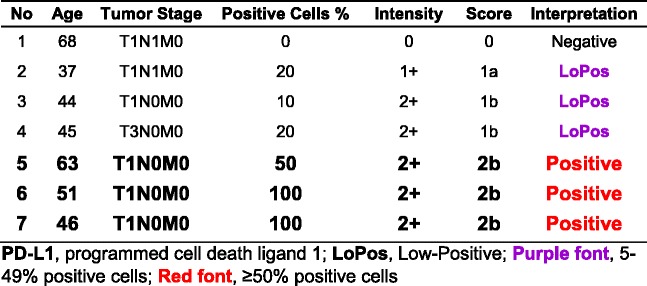
Subgroup IA, summary of cases with **grade-I** squamous cell carcinoma and tumor cell PD-L1 scoring as described in this study

### Subgroup IB, SCC Grade-II

**Fig. 2:**
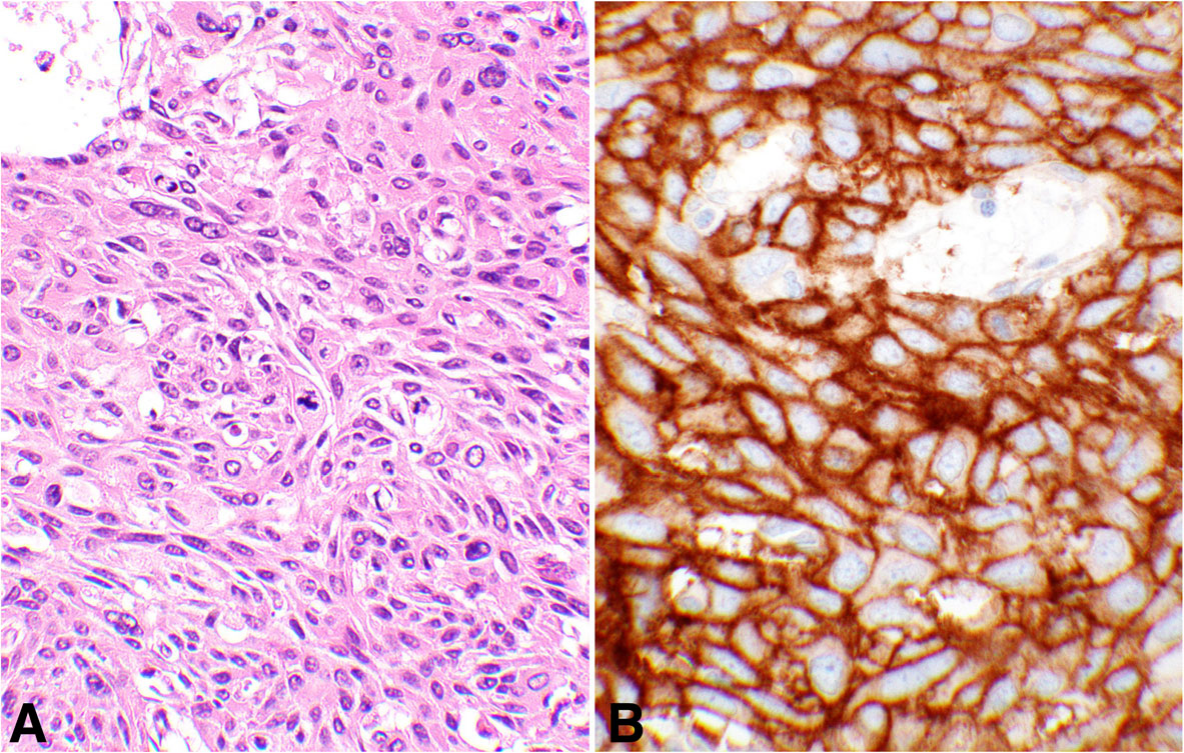
Score 2 in squamous cell carcinoma. An example of Score 2b in grade-II squamous cell carcinoma of the cervix showing PD-L1 staining of 100% of the tumor cells (#49, Table 2). **a** shows hematoxylin and eosin stain of the carcinoma with moderate differentiation containing a mitotic figure at the center of the photomicrograph (objective 40×). **b** demonstrates the intense membrane reaction for PD-L1 with 3+ intensity involving all tumor cells (objective 40×)

This subgroup comprised of 52 core samples with patients’ ages ranging from 30 to 67 with a median of 44.5 years old. Twenty-four (46.2%) of the cases had “Positive” PD-L1 expression, four with the score of “2a” and 20 with the score of “2b”. The rest were nine “Negative,” eight “LoPos, 1a”, and 11 “LoPos, 1b” cores (Table 2). An example of grade-II squamous cell carcinoma with the corresponding positive PD-L1 stain is shown in Fig. 2.

**Table 2:**
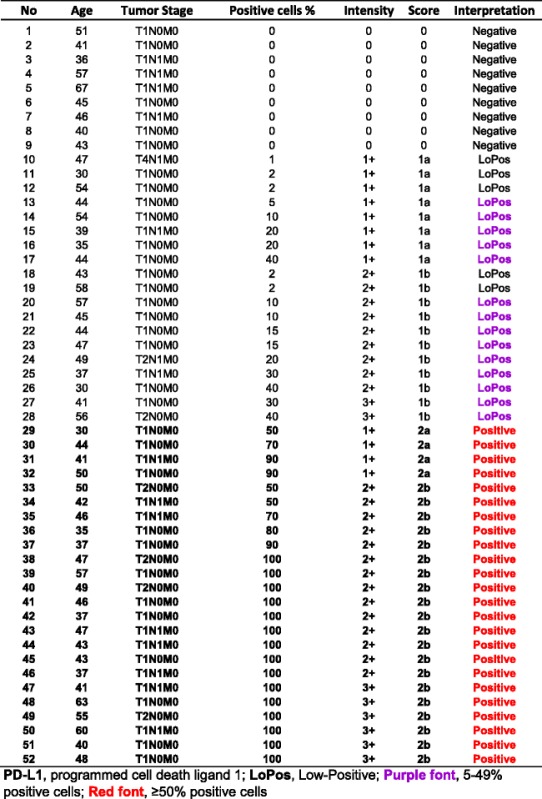
Subgroup IB, summary of cases with **grade-II** squamous cell carcinoma and tumor cell PD-L1 scoring as described in this study

### Subgroup IC, SCC Grade-III

This subgroup comprised of 15 core samples with patients’ ages ranging from 30 to 59, with a median of 39 years old. One (6.7%) case had “Positive” PD-L1 expression with the score of “2b”. The remainder showed six “Negative”, one “LoPos, 1a”, and seven “LoPos, 1b” samples (Table 3).

**Table 3:**
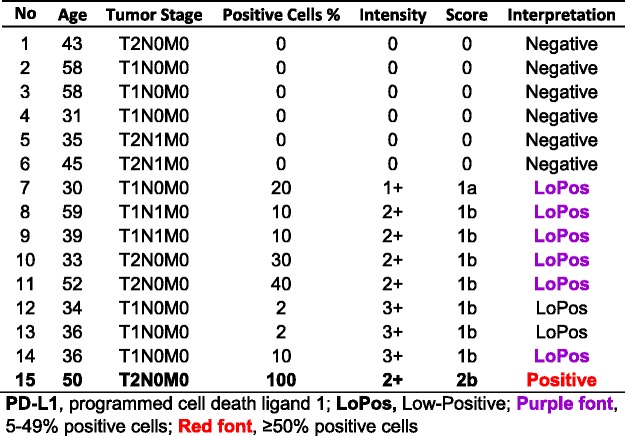
Subgroup IC, summary of cases with grade-III squamous cell carcinoma and tumor cell PD-L1 scoring as described in this study

### Group II, adenosquamous carcinoma

**Fig. 3:**
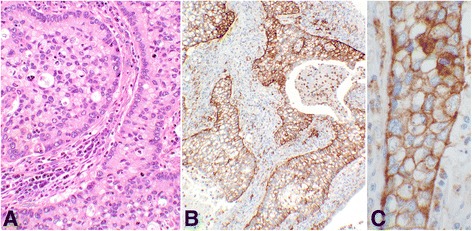
Score 2 in adenosquamous carcinoma. An example of Score 2b adenosquamous carcinoma showing the PD-L1 reaction involving 100% of the tumor cells (#6, Table 4). **a** shows hematoxylin & eosin stain of the glandular organization of the tumor cells with adjacent stromal tissue. The center of the glandular structures contains solid nests of cells with squamoid differentiation (objective 20×). **b** demonstrates the PD-L1 membrane staining of all tumor cells (objective 10×). **c** shows complete circumferential cell membrane staining of the tumor cells with 2+ intensity (objective 40×). This intensity may be interpreted as 3+ by some other pathologists

This group comprised of seven core samples with patients’ ages ranging from 27 to 56, with a median of 44 years old. Two (28.6%) cases had “Positive” PD-L1 expression, one with the score of “2a” and the other with score of “2b”. Remaining five included four “Negative” and one “LoPos, 1b” cores (Table 4). An example of adenosquamous carcinoma with the corresponding positive PD-L1 stain is shown in Fig. 3.

**Table 4:**
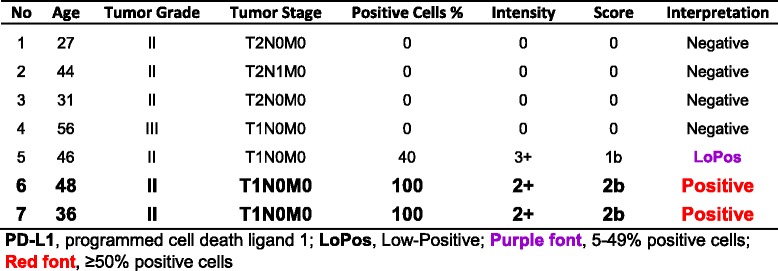
Group II, summary of cases with adenosquamous carcinoma and tumor cell PD-L1 reactions with the final scoring as described in this study

### Group III, endocervical adenocarcinoma

**Fig. 4:**
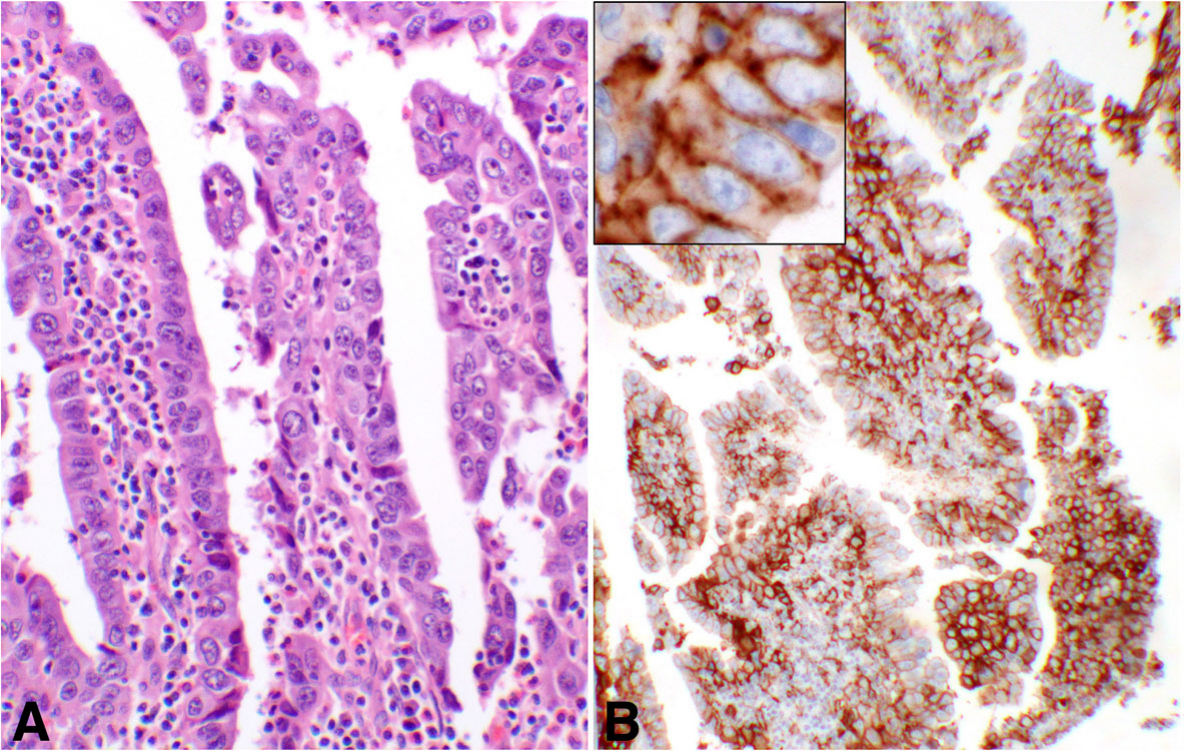
Score 2 in endocervical adenocarcinoma. An example of Score 2b endocervical adenocarcinoma of the cervix with PD-L1 reaction involving 90% of the epithelial tumor cells (#12, Table 5). **a** shows the hematoxylin & eosin stain of the glandular structures with atypical epithelial cell lining and inflammatory stromal core (objective 20×). **b** demonstrates the membrane reaction for PD-L1 with 2+ intensity involving the epithelial tumor cells, many at cross section (objective 10×). The inset shows the circumferential membrane staining of the tumor cells for PD-L1 at a higher magnification (objective 60×)

This group comprised of 12 core samples with patients’ ages ranging from 24 to 50, with a median of 39.5 years old. Two (16.7%) cases had “Positive” PD-L1 expression, one with the score of “2a” and another with a score of “2b”. Remaining ten contained four “Negative”, two “LoPos, 1a”, and three “LoPos, 1b” samples (Table 5). An example of endocervical adenocarcinoma with the corresponding positive PD-L1 stain is shown in Fig. 4.

**Table 5:**
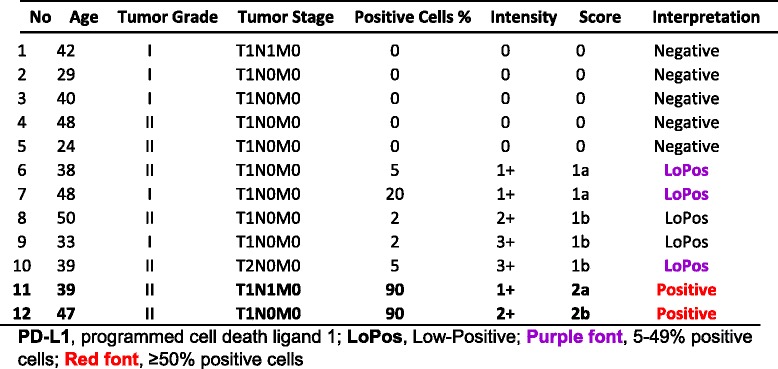
Group III, summary of cases with endocervical adenocarcinoma and tumor cell PD-L1 reactions with the final scoring as described in this study

The findings in the above groups are summarized in Table 6 and show a propensity of “Positive” reaction among squamous cell carcinomas, though, the positivity ranged from 6.7% in grade-III SCC to more than 40% in grade-I and grade-II SCCs. Endocervical adenocarcinomas had the lowest positivity (16.7%) among the three groups. There was no difference between grade-I and grade-II SCCs (*p*-Value = 1), namely the two subgroups were virtually identical (Table 6), therefore lumped together for further analyses. The Fisher Exact statistical analysis yielded a *p*-value of 0.0062 when lower grades (grade I & II) SCCs were tested against high grade (grade III) SCCs (Table 6). When SCCs (all grades) were tested against adenosquamous carcinomas and endocervical adenocarcinomas, *p*-values of 1 and 0.2 were obtained respectively, indicating no statistical differences (Table 6). Even, when the high grade (grade III) SCCs were excluded from the comparison lists, adenosquamous carcinomas and endocervical adenocarcinomas maintained a statistically insignificant difference (*p*-Values of 0.4 & 0.1 respectively) with lower grades SCCs (Table 6).

**Table 6:**
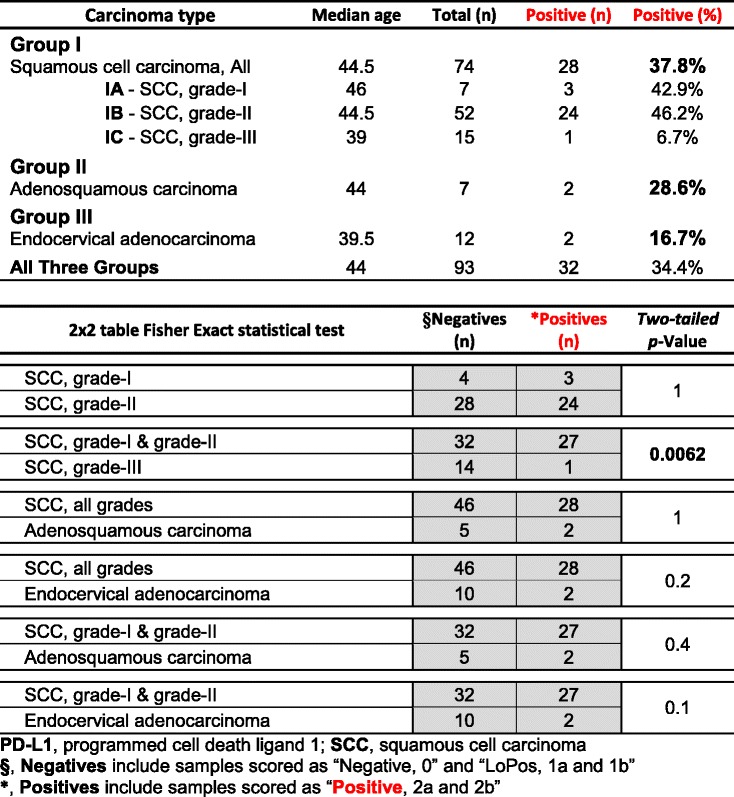
Summary of the cases with Score-3 PD-L1 positivity in the different groups and subgroups of the uterine cervical carcinomas

ᅟ

## Discussions

This study was carried out on a TMA which is made of small size samples. Due to heterogeneity of the PD-L1 reactions, some of the negative samples may have been randomly selected from the “negative” portion of otherwise “positive” tumors. The small size of the TMA samples, however, may mimic the size of the needle biopsy specimens obtained in advanced cancer cases which may have similar PD-L1 reaction outcome. The results of the current study provide a baseline information for the future investigations.

Since there is no unified scoring system for the PD-L1 reactions by IHC, the scoring introduced in this study is based on the Garon et al. NSCLC study [13]. using the antibody clone 22c3 which is used in the Dako platform [21]. In this platform, the cutoff point is set at 50% for the positive cells regardless of the intensity of the reaction. The Ventana platform, based on clone SP142, uses 5% cutoff for positivity, again regardless of the intensity [19]. Food and Drug Administration (FDA) has approved both platforms. Other investigators have used different scoring schemes. In a recently published study, the investigators have used different cutoff points for different immunotherapeutic agents, regardless of the intensity, as listed in the supplemental eTable 3 embedded with their publication [22]. This pathology scoring system faces an issue. As new therapeutic agents are introduced and/or future clinical studies result in changes of the response rates, the scoring system needs to be accordingly adjusted. Therefore, a pathology scoring system is needed for a comprehensive and consistent evaluation of the PD-L1 reactions. Thus, it becomes incumbent upon the clinical oncologist to adjust their protocols as new therapeutic agents are introduced or therapeutic outcomes are changed. The pathologists, however, may amend their scoring reports with comments to include the current rates of the positivity in relation to responses to various immunotherapeutic agents. Although, the 50% cutoff for positivity is employed in this study, the variable rates are also incorporated as “Low-Positive” if less than 50% positive cells are encountered. In this study, we have treated the “Low-Positive” samples as “Negative” for statistical calculations. In addition, the intensity of the IHC reactions is retained as a component of the scoring system for future evaluations.

At the outset, PD-L1 is “positively” expressed in more than 34% of the uterine cervical carcinomas based on the proposed scoring. Its highest expression appears in squamous cell carcinomas although it is significantly lower in the high grade (grade-III) SCCs (Table 6). While the percentages of the “Positive” PD-L1 expression in adenosquamous carcinomas (~29%) and endocervical carcinomas (~17%) are lower than SCCs (~38%), the differences are not statistically significant (Table 6). In other words, PD-L1 is highly expressed in the uterine cervical carcinomas except for the high grade SCC. In all, more than 34% of the patients with uterine cervical cancers may become clinically eligible for the immunotherapy. In addition, about 39% of the patients, in “LoPos, 1a” and “LoPos, 1b” categories may also be considered for the therapy, though a smaller percentage of such cases may respond to the targeted therapy as it has been noted in the NSCLC clinical trial [13].

Therapeutic targeting of the PD-1/PD-L1 immune regulatory axis has led to meaningful results in the treatment of many solid tumors, including melanoma, renal cell carcinoma, NSCLC, and head & neck squamous cell carcinoma [7]. For the treatment of advanced cervical cancer, there are also ongoing clinical phase I/II trials evaluating the effects of anti-PD-1 therapies, including pembrolizumab (NCT02054806) and nivolumab (NCT02488759), although study results have not yet been reported [10]. While data on the use of PD-L1 tumor expression as a screening marker to identify patients for anti-PD-L1 immunotherapies have been conflicting, several studies have found positive correlations between tumor expression of PD-L1 and response to the targeted therapies [11, 23].

Though the use of anti-PD-1/PD-L1 immunotherapies has not been extensively explored for the treatment of cervical cancer, recent studies have evaluated biologic rationales for PD-L1 expression in the cervical tumors, including its pathogenesis through human papillomavirus (HPV), its tumor microenvironment composed of tumor-infiltrating lymphocytes, as well as its genetic basis for increased expression.

Human papilloma virus (HPV) plays a key role in the development of the two most common histologic subtypes of cervical cancer, SCC and endocervical adenocarcinoma. In particular, HPV16 has been linked to 59 and 36%, and HPV18 to 13 and 37%, of SCC and the adenocarcinoma, respectively [24]. The two histologic subtypes have also been found to demonstrate distinct molecular profiles, though current treatment guidelines are generally not type-specific [25]. Some studies have speculated that HPV may activate PD-1/PD-L1 immunosuppression to evade host immune responses against the virus, resulting in persistence and recurrence of cervical intraepithelial neoplasia (CIN) [26]. A recent study by Mezache et al. examined PD-L1 expression in CIN and cervical SCC, and found PD-L1 expression to be strongly associated with HPV infection. In their study, PD-L1 was also upregulated in both the carcinoma and the surrounding inflammatory tissue cells mostly CD8+ lymphocytes [27].

While the current study has evaluated PD-L1 expression of the tumor cells, it is unclear whether PD-L1 expression in tumor-infiltrating inflammatory cells also plays a relevant role in predicting response to anti-PD-L1 therapies [23]. Indeed, some studies have suggested that PD-1/PD-L1 expression in the tumor microenvironment may be important for therapeutic activity [5, 23]. The tumor microenvironment of cervical cancers has been well-studied, with some studies demonstrating the presence of tumor-infiltrating lymphocytes (TILs), and particularly CD8+ T-cells, to be strongly associated with improved clinical outcome and prognosis [28, 29]. A study by Karim et al. found PD-L1 to be expressed in only 19% of cervical cancers, whereas more than half of tumor-infiltrating CD8+ T-cells were positive for PD-1 [30]. Differences in expression from their study may be a result of using a different antibody clone (clone 5H1). Yang et al. also evaluated PD-1 and PD-L1 expression on cervical T-cells and dendritic cells, respectively, and found their upregulation to be associated with high risk-HPV positivity and increasing CIN grade [31]. Together, these findings suggest a strong immunopathogenic rationale for PD-L1 expression in cervical carcinomas.

Several studies have explored a genetic basis for PD-L1 expression in various cancers, as well as its correlation with clinical outcome. In particular, *PD-L1* amplification and deletion has been found to be associated with poor overall survival amongst many major cancer types [32]. Overexpression of PD-L1 by immunohistochemical methods has also been found to be associated with worse survival in solid tumors, including esophageal cancer, gastric cancer, colorectal cancer, and pulmonary adenocarcinoma [8, 9]. Ironically, a higher percentage of the reaction has occurred in the lower grade carcinomas in this study. This finding may be due to a lower sample size of grade III SCCs in this series and/or the scoring which has excluded the “Low-Positive” tumors as “Positive” (Table 3). Besides, clone SP142 yields a lower number of positive cells by IHC [22], however, it is not clear which clone is most relevant to the clinical outcome of cervical carcinomas. Further controlled studies are required to elucidate the clone-outcome relationships in uterine cervical cancers. In a study regarding uterine cervical cancers, the investigators have lumped adencarcinomas and SCCs as a sinle group with no tumor grading distiction [10]. There is, however, a general agreement that a higher expression of PD-L1 is associated with a poorer prognosis in uterine cervix, endometrium, and head & neck caners [10, 33–35].

As recent trials have suggested tumors, with a genetic basis for PD-1 ligand expression, are particularly sensitive to anti-PD-1 immunotherapies. Howitt et al. have investigated the status of genes encoding PD-1 ligands in cervical SCC. They have found co-gain or co-amplification of *CD274* and *PDCD1LG2,* coding PD-L1 and PD-L2, in a significant number of cervical SCCs [36]. In a recent study of cervical SCC, patients with diffuse expression of PD-L1 were also found to have significantly worse disease-free and disease-specific survival as compared to those with only marginal PD-L1 expression [10].

Finally, the scoring algorithm in this study has been adopted from the non-small cell lung cancers study [13], noting that NSCLCs and cervical carcinomas are the same entity. Our study has been carried out on a TMA with little clinical information and no treatment data. Therefore, the cutoff points for positivity for each therapeutic agent should be determined after the clinical trials for cervical cancers have been completed. Until then, the adopted scoring is merely a reporting mean for pathologists in which 50% as well as variable cutoffs are integrated into a single system with addition of the IHC intensities.

## Conclusions

This study has found a significant expression of PD-L1 in cervical malignancies, and no expression of PD-L1 in normal cervix or benign cervical lesions. The current literature has identified potential genetic and immunopathogenic rationales for PD-L1 expression in cervical cancers. The findings in this study further support a role for the investigation of anti-PD-L1/PD-1 immunotherapies for the treatment of PD-L1-positive cervical tumors. In addition, our adopted scoring system will help to identify different responders to the immunotherapy as correlated with the degree of PD-L1 expression. Thus, we recommend interpretation and reporting of PD-L1 staining to include the adopted score, percentage of the positive cells, and the intensity of the reaction (e.g., ***Positive: 2a, 80%, 1+*** or ***Low-Positive: 1b, 10%, 3+***) to facilitate a more solid correlation with response to the treatment. Future clinical trials, based on response to the targeted immunotherapies, may help to further refine the proposed scoring system.

## Abbreviations

HPV: Human papillomavirus
IHC: Immunohistochemistry
LoPos: Low-Positive
NSCLC: Non-small cell lung carcinomas
PD-1/PD-L1: Programmed death-1/programmed death-ligand1
SCC: Squamous cell carcinoma
TMA: Tissue microarray
